# Exercise as a therapeutic strategy for depression in menopausal women: a metaanalysis of randomized trials

**DOI:** 10.3389/fpsyt.2025.1641082

**Published:** 2025-09-19

**Authors:** Sen Li, Yan Dou, Ye Li

**Affiliations:** ^1^ Sports Teaching and Research Department, Heilongjiang Institute of Technology, Harbin, China; ^2^ Department of Sports Training, Hebei Sport University, Shijiazhuang, China; ^3^ St. Paul University Philippines, Cagayan, Philippines

**Keywords:** exercise intervention, menopause, depression, meta-analysis, randomised controlled trials

## Abstract

**Background:**

Menopause is a transitional phase in a woman’s life marked by a heightened vulnerability to depressive symptoms. Exercise has emerged as a promising non-pharmacological strategy for alleviating depression, yet the extent to which different intervention characteristics influence outcomes remains unclear.

**Objective:**

This meta-analysis aimed to evaluate the overall effectiveness of exercise interventions in reducing depressive symptoms among menopausal women and to examine potential moderators through detailed subgroup analyses.

**Methods:**

A comprehensive search of four databases identified 16 randomised controlled trials (RCTs) meeting the inclusion criteria. Standardised mean differences (SMDs) were calculated to quantify effect sizes. Subgroup analyses were conducted based on exercise format (individual vs. group), exercise type, session length, total intervention duration, and menopausal stage. Sensitivity analysis and Egger’s test were used to assess result stability and publication bias, respectively.

**Results:**

Exercise interventions were associated with a significant reduction in depressive symptoms (SMD=–1.04, 95% CI: –1.46 to –0.63, p < 0.00001). Subgroup analyses indicated that individual-based formats, mind-body exercises (e.g., yoga, tai chi), longer sessions (60–90 min), extended intervention durations (>12 weeks), and interventions during the perimenopausal stage produced greater effects. Egger’s test suggested no significant publication bias (p=0.441), and sensitivity analyses confirmed the robustness of the findings.

**Conclusion:**

Exercise is an effective intervention for reducing depressive symptoms in menopausal women. The magnitude of benefit varies by intervention characteristics, underscoring the need for personalised, phase-specific exercise prescriptions. These findings provide a strong evidence base for integrating structured exercise into mental health strategies targeting midlife women.

**Systematic Review Registration:**

https://www.crd.york.ac.uk/prospero/, identifier CRD420251046026.

## 1 Background

Depression is one of the most prevalent and disabling mental health disorders worldwide (1, 2). According to the World Health Organization, approximately 280 million people globally are affected. Core symptoms include persistent low mood, anhedonia, cognitive impairment, and somatic complaints (3). These symptoms substantially diminish quality of life and are associated with increased risks of social dysfunction, physical morbidity, and suicide (4, 5). Notably, the burden of depression is unequally distributed by gender, with women experiencing significantly higher rates than men—an imbalance attributed to distinct physiological and psychosocial stressors encountered throughout life (6).

Menopause represents a critical window of vulnerability for depression onset in women (7, 8). Defined as the biological transition marked by declining ovarian function and the cessation of menstruation—typically occurring between ages 45 and 55—this period is associated with pronounced hormonal fluctuations and a constellation of physiological and psychological symptoms, including sleep disturbances, vasomotor symptoms, and emotional instability (9, 10). A substantial body of evidence has demonstrated that both perimenopausal and postmenopausal women face an elevated risk of depression (11). While endocrine changes are central, sociocultural stressors such as shifting familial roles, caregiving burdens, and diminished social support also play key roles (12, 13).

It is important to distinguish between physical activity and exercise. Physical activity is broadly defined as any bodily movement produced by skeletal muscles that requires energy expenditure. In contrast, exercise is a more specific subset of physical activity that is planned, structured, and repetitive, and performed with the objective of improving or maintaining physical fitness. In recent years, exercise has emerged as a promising non-pharmacological intervention for depression (14). A growing body of literature suggests that regular exercise can mitigate depressive symptoms through mechanisms involving neurochemical modulation, improved sleep quality, and enhanced self-efficacy (15, 16). For individuals reluctant to use or unresponsive to antidepressants, exercise offers a low-risk alternative. Evidence from studies involving menopausal women indicates that modalities such as aerobic exercise, resistance training, tai chi, and yoga may confer psychological benefits (17, 18). However, the literature remains heterogeneous, and the influence of intervention characteristics—such as mode, frequency, and duration—warrants further synthesis.

Given the rising incidence of depression among menopausal women and the expanding research on exercise-based interventions, this study is both timely and necessary. Although several randomised controlled trials (RCTs) have investigated the therapeutic potential of exercise in this population, comprehensive meta-analyses consolidating these findings remain limited. This study aims to systematically review and quantitatively evaluate the effects of exercise interventions on depressive symptoms in menopausal women. In doing so, it also seeks to identify key moderating variables, providing evidence-based guidance for the development of tailored, scientifically grounded intervention strategies.

## 2 Methods

This meta-analysis was performed according to the Preferred Reporting Items for Systematic Reviews and Meta-Analysis statement and the Cochrane Collaboration Handbook. The protocol was registered on PROSPERO (CRD420251046026).

### 2.1 Data sources and searches

The systematic search was conducted by two independent reviewers (LS and DY) in four databases: the Cochrane Library, Embase, PubMed and Web of Science, and was designed to retrieve articles up to April 2025, with disagreements resolved by consensus and by a third reviewer (LY) in case of disagreement. Terms from the Medical Subject Headings (MeSH) and words from the text were used as follows: (“Menopause” OR “Change of Life, Female”) AND (“Depression” OR “Depressive Symptoms” OR “Depressive Symptom” OR “Symptom, Depressive” OR “Emotional Depression” OR “Depression, Emotional”) AND (“Exercise” OR “Exercises” OR “Physical Activity” OR “Activities, Physical” OR “Activity, Physical” OR “Physical Activities” OR “Exercise, Physical” OR “Exercises, Physical” OR “Physical Exercise” OR “Physical Exercises” OR “Acute Exercise” OR “Acute Exercises” OR “Exercise, Acute” OR “Exercises, Acute” OR “Exercise, Isometric” OR “Exercises, Isometric” OR “Isometric Exercises” OR “Isometric Exercise” OR “Exercise, Aerobic” OR “Aerobic Exercise” OR “Aerobic Exercises” OR “Exercises, Aerobic” OR “Exercise Training” OR “Exercise Trainings” OR “Training, Exercise” OR “Trainings, Exercise”) Specific details of the search algorithms for each database are provided in **Supplementary Material 1**.

### 2.2 Inclusion and exclusion

This study was conducted in accordance with the PICOS framework of the Cochrane systematic review methodology. The following inclusion criteria were strictly defined: **(P)** Participants were women in the peri-menopausal or postmenopausal stages, generally aged 45 years or older, and explicitly identified as menopausal in the original studies. **(I)** The intervention comprised a single form of exercise, such as aerobic training, resistance training, tai chi, or dance, with clearly reported frequency, intensity, and duration. **(C)** The comparator group received no intervention, routine health education, a waitlist condition, or other non-exercise-based interventions. **(O)** Outcomes were limited to those assessing changes in depressive symptoms using validated, standardised measurement tools, including the Beck Depression Inventory (BDI), Hospital Anxiety and Depression Scale (HADS), Geriatric Depression Scale (GDS), Self-Rating Depression Scale (SDS), the mental health component of the 36-Item Short Form Health Survey (SF-36), General Health Questionnaire-28 (GHQ-28), Symptom Checklist-90-Revised (SCL-90-R), Patient Health Questionnaire-8 (PHQ-8), and Brief Symptom Inventory-18 (BSI-18). **(S)**Only published randomised controlled trials (RCTs) with full-text availability were included.

The following exclusion criteria were applied during the screening process: (1) studies involving participants who were not peri- or postmenopausal women; (2) comparator groups that did not provide a valid baseline for comparison; (3) interventions combining multiple treatment modalities or using non-exercise strategies, precluding isolation of exercise effects; (4) insufficient or unextractable data on intervention protocols or outcomes; (5) studies lacking outcome indicators specifically related to depressive symptoms; (6) protocols, pilot studies, or registration records without reported results; and (7) study populations with significant physical or mental comorbidities that could confound depression assessments.

### 2.3 Assessment of risks of bias

In adherence to the esteemed Cochrane Collaboration guidelines, reviewers LS and DY independently conducted a meticulous risk of bias assessment for each study included in our analysis. The Cochrane Collaboration’s guidelines provide a comprehensive and internationally recognized framework, allowing for a standardized evaluation of each study’s methodological rigor. This rigorous assessment process, rooted in the Cochrane ethos, involves a detailed examination of potential biases, including random sequence generation, allocation concealment, blinding of participants and personnel (a particularly complex issue in behavioural interventions), completeness of outcome data, selective reporting, and other sources of bias. All evaluations were independently performed by the two reviewers, with any discrepancies resolved through discussion or, if needed, consultation with a third reviewer (LY). The results of this thorough risk of bias assessment are systematically compiled in the Risk of Bias Table, presented in **Supplementary Material 2**, offering a transparent and comprehensive summary of the methodological quality of each included study. Importantly, in interpreting the meta-analytic findings and drawing conclusions about the efficacy of exercise interventions for depression in menopausal women, these risk of bias assessments were carefully taken into account. This process ensures a balanced, evidence-based interpretation aligned with the high standards of methodological integrity championed by the Cochrane Collaboration.

### 2.4 Data extraction

Using a standardized data extraction form, two reviewers (LS and DY) independently extracted relevant information from each included study. The extracted data encompassed key study characteristics, including author names, year of publication, sample sizes of the intervention and control groups, age-related characteristics of participants in both groups, type of intervention, intervention duration, frequency, and session length, nature of the control group, and outcome measures. The use of a standardized form ensured consistency and accuracy across the data extraction process, thereby minimizing potential errors and enhancing the reliability of the dataset.

Each reviewer systematically recorded the required information, and any discrepancies or uncertainties were resolved through discussion or, when necessary, by consulting a third reviewer (LY). This rigorous and methodical approach to data extraction enabled the acquisition of comprehensive and trustworthy data from the eligible studies, which served as the empirical foundation for the subsequent meta-analysis. The complete set of extracted data is provided in the supplementary materials, offering transparency and facilitating a clear understanding of the fundamental characteristics of the studies included in this review.

### 2.5 Assessment of overall effect size

Statistical analyses were performed using Review Manager (RevMan) version 5.3. A total of sixteen studies were included in the meta-analysis, and overall effect sizes were calculated based on the statistical results derived from standardized depression measurement scales. To assess the magnitude of the intervention effects, Hedges’ g standardized effect sizes were computed for each study, with values of 0.2, 0.4, and 0.8 conventionally interpreted as small, medium, and large effects, respectively. All effect sizes were adjusted to ensure they reflected the expected direction of the intervention, and statistical significance was defined as p < 0.05. Given the variability in depression outcome measures across studies on menopausal women, the standardized mean difference (SMD) was employed as the effect size metric, allowing for the synthesis of results across different scales. The SMD and corresponding 95% confidence intervals (CIs) were calculated using the Practical Meta-Analysis Effect Size Calculator (Wilson). To evaluate between-study variability, heterogeneity was assessed using the Q statistic and the I² index. I² values of 0% indicated no heterogeneity, ≥25% low, ≥50% moderate, and ≥75% high heterogeneity. In cases of moderate to high heterogeneity, a random-effects model was applied to account for inter-study variability; otherwise, a fixed-effects model was used to pool the results. This statistical approach ensured a robust and comprehensive evaluation of the effect of exercise interventions on depression among menopausal women.

### 2.6 Subgroup analysis of exercise intervention programmes

To further elucidate the effects of exercise intervention characteristics on the reduction of depressive symptoms in postmenopausal women, five predefined subgroup analyses were conducted. These analyses were based on both intervention modalities and participant characteristics. First, to examine the potential moderating role of social engagement, exercise interventions were categorised as either individual-based or group-based, reflecting the organisational structure of participation. Second, interventions were stratified by type of exercise, including aerobic activity, mind–body practices (e.g., yoga, tai chi), and resistance training, in order to compare differential impacts across modalities. Third, the total intervention duration was analysed, grouping studies into ≤12 weeks and >12 weeks to assess temporal influences on treatment efficacy. Fourth, exercise sessions were categorised by per-session duration (i.e., 30–60 minutes, 60 minutes, 60–90 minutes) to evaluate potential dose-response relationships. Finally, subgroup analyses were performed based on menopausal status (perimenopausal vs. postmenopausal) to investigate differential responsiveness across hormonal transition stages.

The included studies were stratified by the type of exercise intervention. We classified interventions as ‘exercise’ only if they were structured, planned, and met specific criteria such as a defined frequency (e.g., three times per week), duration (e.g., 60 minutes per session), and intensity (e.g., moderate intensity). For instance, walking was classified as ‘aerobic exercise’ only when it was prescribed with a specific structure and intensity, rather than as general physical activity. Similarly, mind-body practices like yoga or tai chi were included as ‘exercise’ if they were part of a structured program designed to improve health outcomes.

For all subgroup analyses, relevant data were extracted from the included studies, and effect sizes (Hedges’ g) with 95% confidence intervals were calculated using Review Manager v5.3. A significance threshold of p < 0.05 was applied, and between-subgroup heterogeneity was assessed using standard statistical tests. These analyses aim to refine our understanding of how specific features of exercise programmes influence depressive symptomatology in menopausal women and to inform tailored intervention strategies. The findings from the subgroup analyses are presented in detail and further interpreted in the Discussion section to guide future clinical and research applications.

## 3 Results

### 3.1 Search process

A total of 977 records were identified through database searches. After removing 52 duplicates, 925 records remained for initial screening. Title-based screening led to the exclusion of 744 articles deemed irrelevant, followed by the removal of 12 systematic reviews or meta-analyses. As a result, 169 full-text articles were assessed for eligibility.

**Figure 1:**
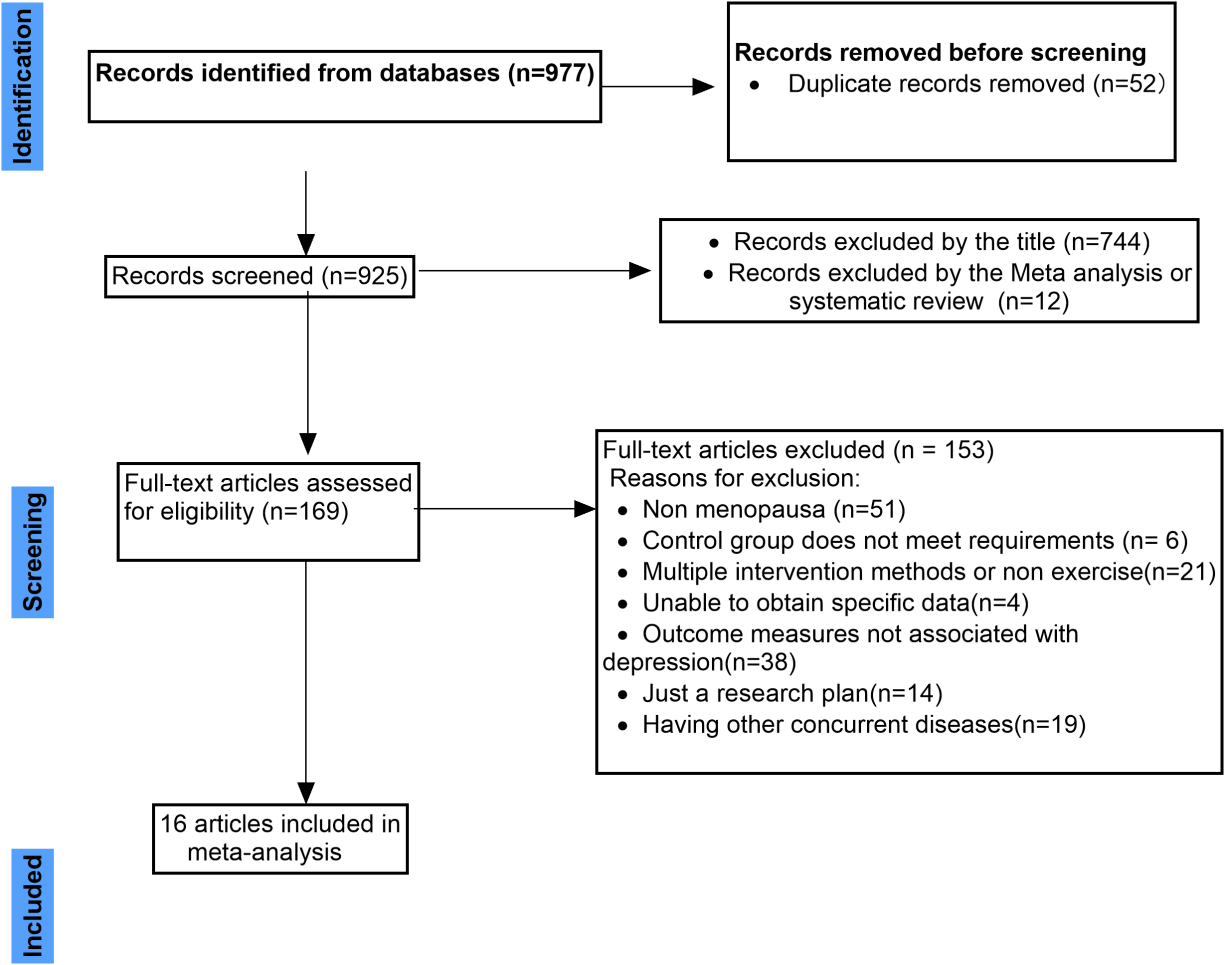
Flowchart and selection of studies.

Among these, 153 studies were excluded for the following reasons: the study population did not include perimenopausal or postmenopausal women (n=51); the control group design was inadequate (n=6); the intervention involved multiple components or non-exercise elements, precluding isolation of exercise effects (n=21); intervention or outcome data were unavailable or not extractable (n=4); outcome measures did not assess depressive symptoms (n=38); only study protocols or trial registrations were available with no reported outcomes (n=14); and the presence of significant comorbidities among participants could have confounded depression assessments (n=19).Ultimately, 16 randomised controlled trials met the inclusion criteria and were included in the meta-analysis. The full literature screening process is summarised in **Figure 1**. The activities listed in **Table 1**, such as walking, dancing, and yoga, were all conducted as structured exercise interventions within the context of the included studies.

**Table 1:** Characteristics of the included studies and participants.

Studies	Sample size (IG/CG)	Age range (IG/CG)	Menopausal status	IG type	Frequency/duration	Outcome measures
Abedi et al. (19)	49:48	52.4±3.8/53±4.1	postmenopausal	walking	/12 weeks	BDI
Aibar-Almazán et al. (20)	55:52	69.98±7.83/66.79±10.14	postmenopausal	Pilates exercise	2 times weekly/12 weeks	HADS
Bernard et al. (21)	61:60	65.46±4.37/65.5±4.03	postmenopausal	walking	3 times weekly/6months	BDI
Carcelén-Fraile et al. (22)	57:68	69.70±6.15/69.75±6.76	postmenopausal	qigong	2 times weekly/12 weeks	HADS
Gao et al. (23)	26:24	54.5±4.5/53.5±4.7	perimenopausal	dance	5 times weekly/3months	SDS
Hu et al. (24)	40:40	52.60±4.12/54.15±2.32	postmenopausal	walking	3 times weekly/4 months	BDI
Jorge et al. (25)	40:19	54±6/55±4	postmenopausal	yoga	2 times weekly/12 weeks	BDI
Kai et al. (26)	20:20	51.0±7.0/51.2±7.9	perimenopausal	stretching	7 times weekly/3 weeks	SDS
Martin et al. (27)	95:92	56.5±6.7/57.1±6	postmenopausal	Aerobic Exercise	3 times weekly/6 months	SF-36
Nikkhah et al. (28)	43:41		postmenopausal	walking	3 times weekly/12 weeks	GHQ-28
Noh et al. (29)	21:19	59.38±3.76/58.21±3.99	postmenopausal	walking	3 times weekly/12 weeks	SCL-90-R
Villaverde Gutiérrez et al. (30)	27:30		postmenopausal	Combined exercise	2–3 times weekly/ 6 months	GDS
Sternfeld et al. (31)	78:133	55.8±3.6/54.2±3.5	postmenopausal	Aerobic Exercise	3 times weekly/3 months	PHQ-8
Imayama et al. (32)	117:87	58.1± 5.0/57.4± 4.4	postmenopausal	Aerobic Exercise	5 times weekly/12 months	BSI-18
Liu et al. (33)	32:34	47.2±1.2/47.8±2.5	perimenopausal	Taiji	5 times weekly/12 weeks	SDS
Martins et al. (34)	23:24		postmenopausal	dance	2 times weekly/16 weeks	HADS

IG, Intervention Group; CG, Control Group; BDI, Beck Depression Inventory; HADS, Hospital Anxiety Depression Scale; GDS, Geriatric Depression Scale; SDS, Self-Rating Depression Scale; SF-36, 36-Item Short Form Health Survey; GHQ-28, General Health Questionnaire-28; SCL-90-R, Symptom Checklist-90-Revised; PHQ-8, Patient Health Questionnaire-8; BSI-18, Brief Symptom Inventory-18.

### 3.2 Characteristics of the included studies and participants

The characteristics of the 16 studies included in this meta-analysis are summarised in **Table 1**. Detailed information is provided on sample size, mean participant age, menopausal status, intervention and control group design, type of exercise intervention, intervention duration and frequency, as well as the depression outcome measures used in each study.

### 3.3 Risks of bias

All 16 included studies were judged to have a low risk of bias for random sequence generation. Allocation concealment was adequately reported in 9 studies and rated as low risk, while the remaining 7 studies were assessed as having unclear risk due to insufficient reporting. Participant blinding was associated with high risk of bias in 10 studies. In terms of outcome assessor blinding, 7 studies were judged as low risk, and 3 were rated as unclear risk due to lack of detail.

For incomplete outcome data, 13 studies were assessed as low risk. Selective reporting was judged to be low risk in 5 studies, while 11 were rated as unclear due to limited reporting. Additionally, 13 studies were considered to have an unclear risk of other potential sources of bias. A comprehensive summary of the risk of bias assessment is provided in **Supplementary Material 2**.

### 3.4 Meta-analysis

#### 3.4.1 Baseline period test

The 16 randomised controlled trials included in this meta-analysis employed a range of depression assessment tools (e.g., BDI, HADS, GDS, SDS, SF-36, GHQ-28, SCL-90-R, PHQ-8, BSI-18). To account for differences in measurement scales, the standardised mean difference (SMD) was used to harmonise baseline depression levels across intervention and control groups.

**Figure 2:**
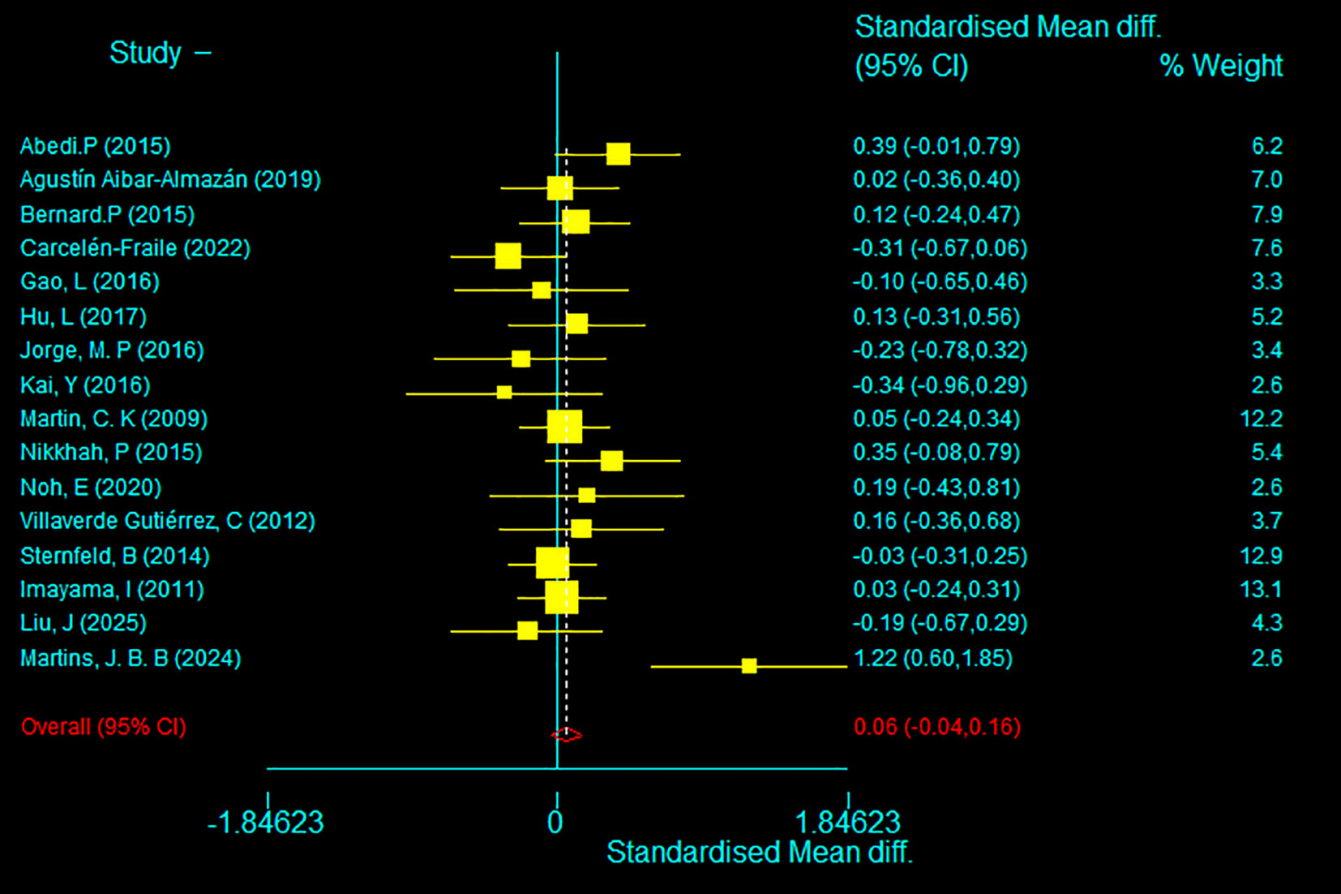
Baseline period test.

Meta-analytic results indicated no significant difference in baseline depression scores between the two groups (SMD=0.06, 95% CI: –0.04 to 0.16; p=0.253), suggesting comparable levels of depressive symptoms prior to intervention. This finding supports the methodological integrity and comparability of subsequent effect size estimations. Detailed baseline consistency results are presented in **Figure 2**.

#### 3.4.2 Meta-analysis result

This meta-analysis included 16 randomised controlled trials that met the predefined inclusion criteria. The effectiveness of exercise interventions in reducing depressive symptoms among postmenopausal women was evaluated using the standardised mean difference (SMD) as the measure of effect size. The pooled analysis demonstrated a significant reduction in depressive symptoms in the intervention group compared with controls (SMD=–1.04, 95% CI: –1.46 to –0.63; p < 0.00001), indicating a robust overall benefit of exercise interventions in this population.

The heterogeneity test revealed substantial between-study variability (I²=93%, χ²=213.87, df=16; p < 0.00001). To assess the robustness of the findings, a sensitivity analysis was conducted by sequentially excluding individual studies. The overall effect size remained stable, and heterogeneity levels showed minimal fluctuation, suggesting that the results are not unduly influenced by any single study.

**Figure 3:**
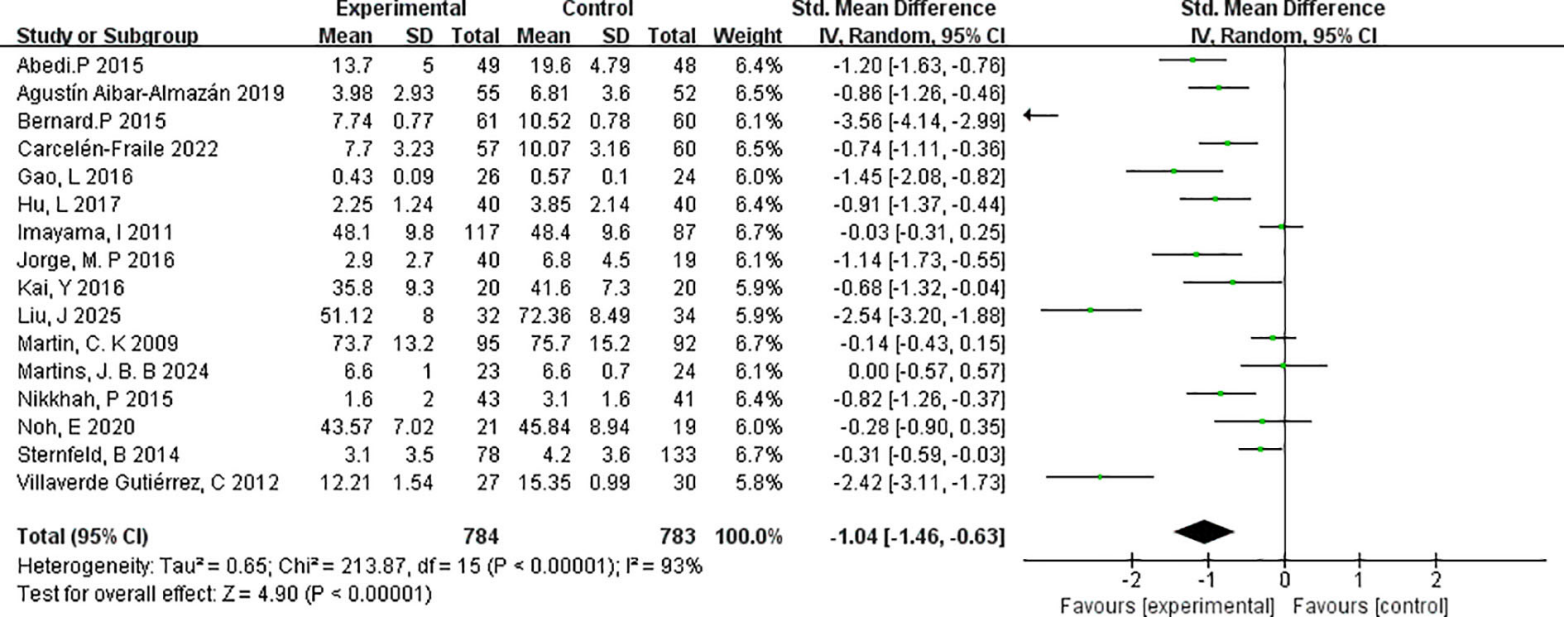
Forest plot of exercise for depression in menopausal women.

It is worth noting that three of the included trials involved perimenopausal rather than postmenopausal women, which may have contributed to the observed heterogeneity. Nevertheless, the beneficial effects of exercise on depressive symptoms were consistent across different stages of the menopausal transition. Despite the high heterogeneity, the analysis revealed a clear and statistically significant effect, supporting the utility of exercise as a non-pharmacological intervention with considerable potential for alleviating depressive symptoms in menopausal women. Full results of the analysis are presented in **Figure 3**.

#### 3.4.3 Publication bias test

**Figure 4:**
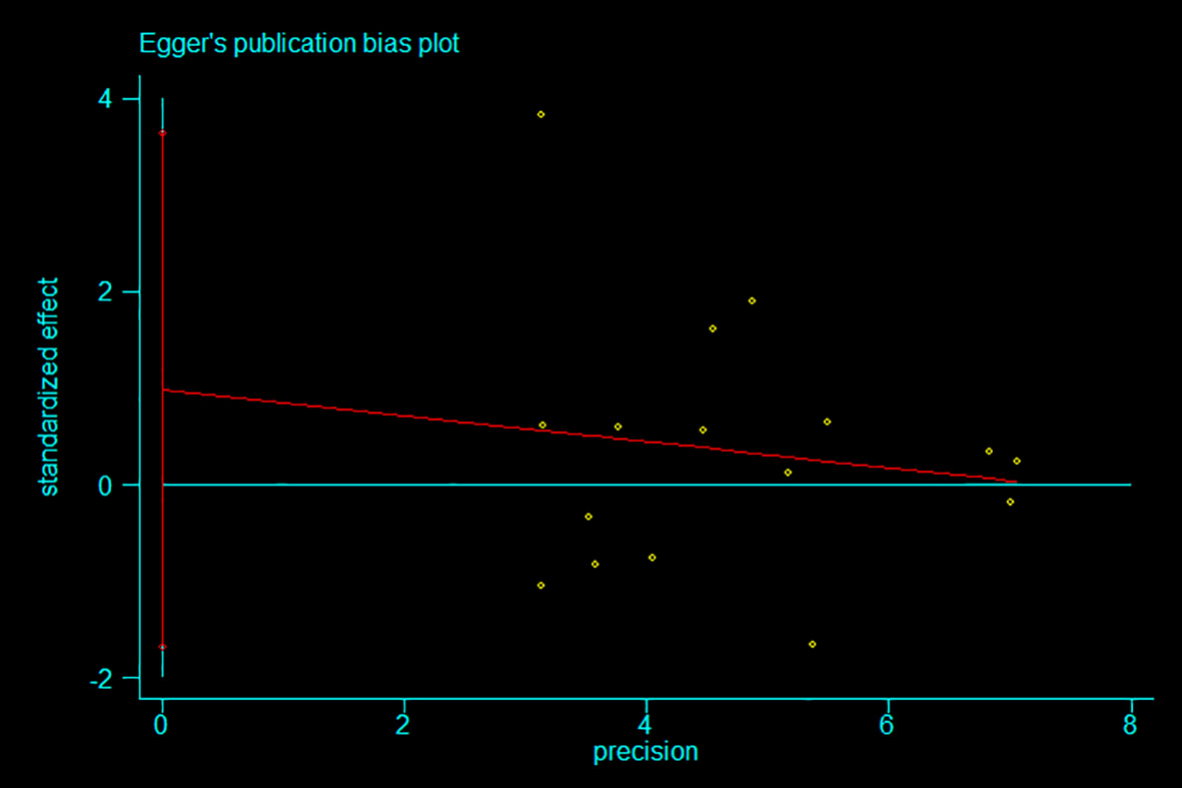
Egger’s publication bias plot.

Publication bias was visually assessed using Egger’s publication bias plot (**Figure 4**). To complement this, we conducted Egger’s test to quantitatively evaluate the potential presence of small-study effects and publication bias.

Egger’s test was selected for its suitability in detecting bias in meta-analyses involving continuous outcomes, such as standardised mean differences (SMDs), which were used in this study. The method evaluates the regression of effect size on its standard error, thereby identifying systematic asymmetry related to study precision. Compared to alternatives such as Begg’s test or funnel plot inspection, Egger’s test is more sensitive in detecting small-study effects—an important consideration given that several included studies had relatively small sample sizes.

While Egger’s test has limitations—such as reduced sensitivity to non-linear trends and susceptibility to distortion in the presence of extreme heterogeneity—these concerns were judged to have limited impact given the structure and characteristics of our dataset. Alternative methods were also considered; however, Egger’s test offered the most appropriate balance of sensitivity and statistical rigour for the present analysis.

**Figure 5:**
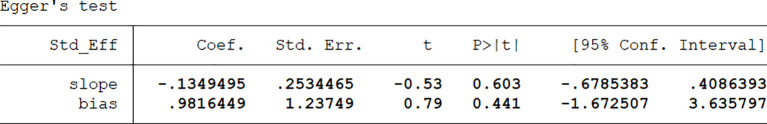
Egger’s test.

The results of Egger’s test (**Figure 5**) indicated no significant evidence of publication bias. The bias coefficient was 0.982 (95% CI: –1.67 to 3.64; p=0.441), and the slope term was also non-significant (p=0.603), supporting the conclusion that small-study effects were not a major source of distortion. These findings suggest that the pooled effect estimates are robust and unlikely to be substantially influenced by publication bias.

#### 3.4.4 Subgroup analysis

Subgroup analyses were conducted to examine five key characteristics of the exercise interventions: exercise format (individual *vs*. group-based), type of exercise, duration of each session, total intervention duration, and menopausal stage of participants.

##### 3.4.4.1 Individual versus group

**Figure 6:**
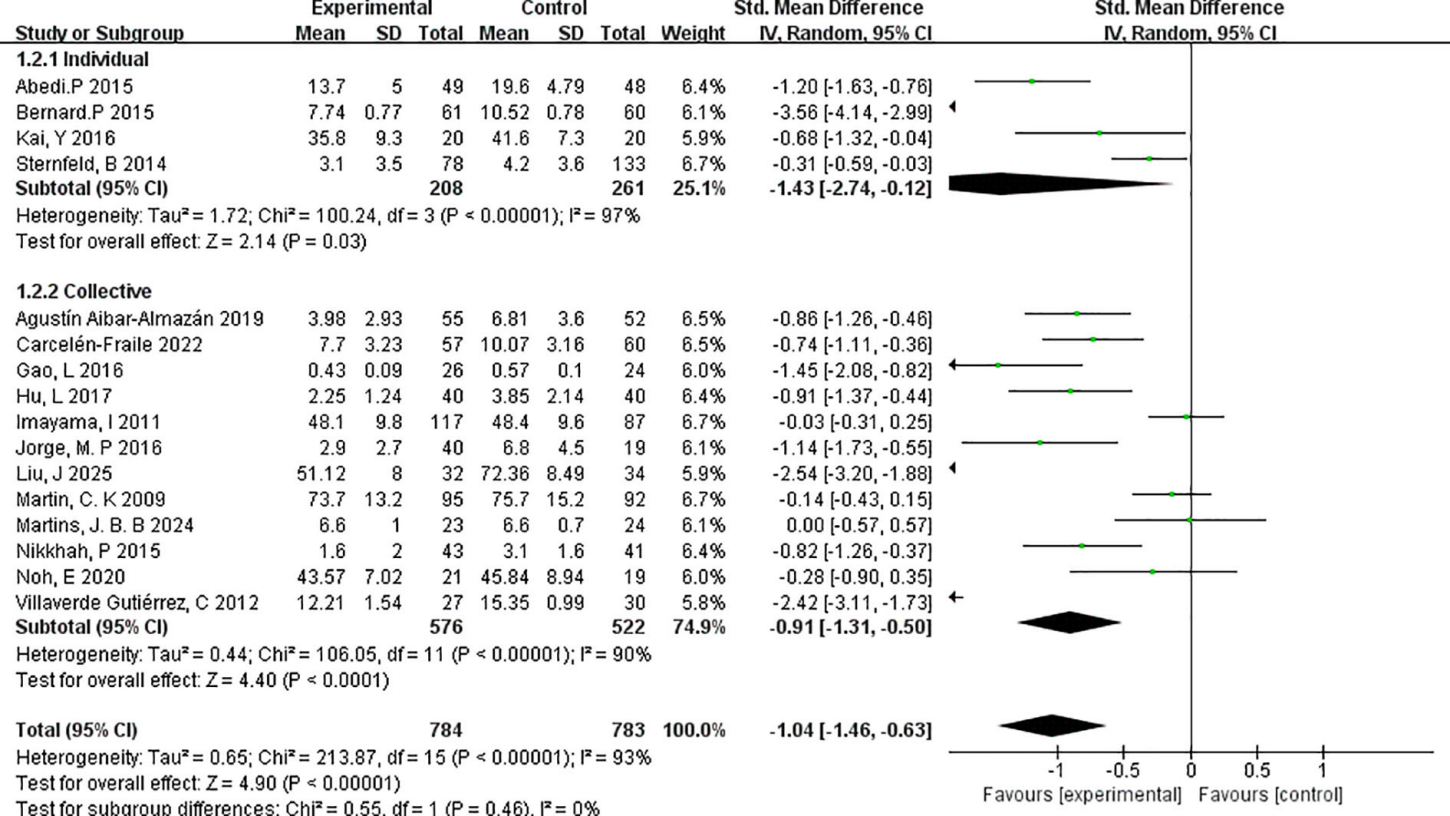
Forest plot of subgroup analyses by individual versus group.

To assess the influence of exercise format on intervention outcomes, a subgroup analysis was performed comparing individual-based versus group-based exercise interventions (**Figure 6**). The analysis revealed that individual exercise interventions yielded a stronger effect in reducing depressive symptoms compared to group-based formats.

Specifically, the individual exercise subgroup (k=4) demonstrated a pooled effect size of SMD=–1.43 (95% CI: –2.74 to –0.12; p=0.03), indicating a large and statistically significant effect. However, substantial heterogeneity was present (I²=97%), suggesting notable variability among studies. The studies in this subgroup included interventions with relatively small sample sizes and varied measurement tools, which may have contributed to the heterogeneity.

In comparison, the group-based exercise subgroup (k=12) showed a significant but slightly smaller effect size of SMD=–0.91 (95% CI: –1.31 to –0.50; p < 0.0001), with moderate heterogeneity (I²=90%). This suggests that while both individual and group exercise formats are effective, individual interventions may confer greater benefits in alleviating depressive symptoms among menopausal women.

These findings underscore the potential value of personalised, one-on-one exercise formats in mental health interventions during menopause. However, the higher heterogeneity in the individual subgroup warrants cautious interpretation and further research.

##### 3.4.4.2 Menopausal stage

**Figure 7:**
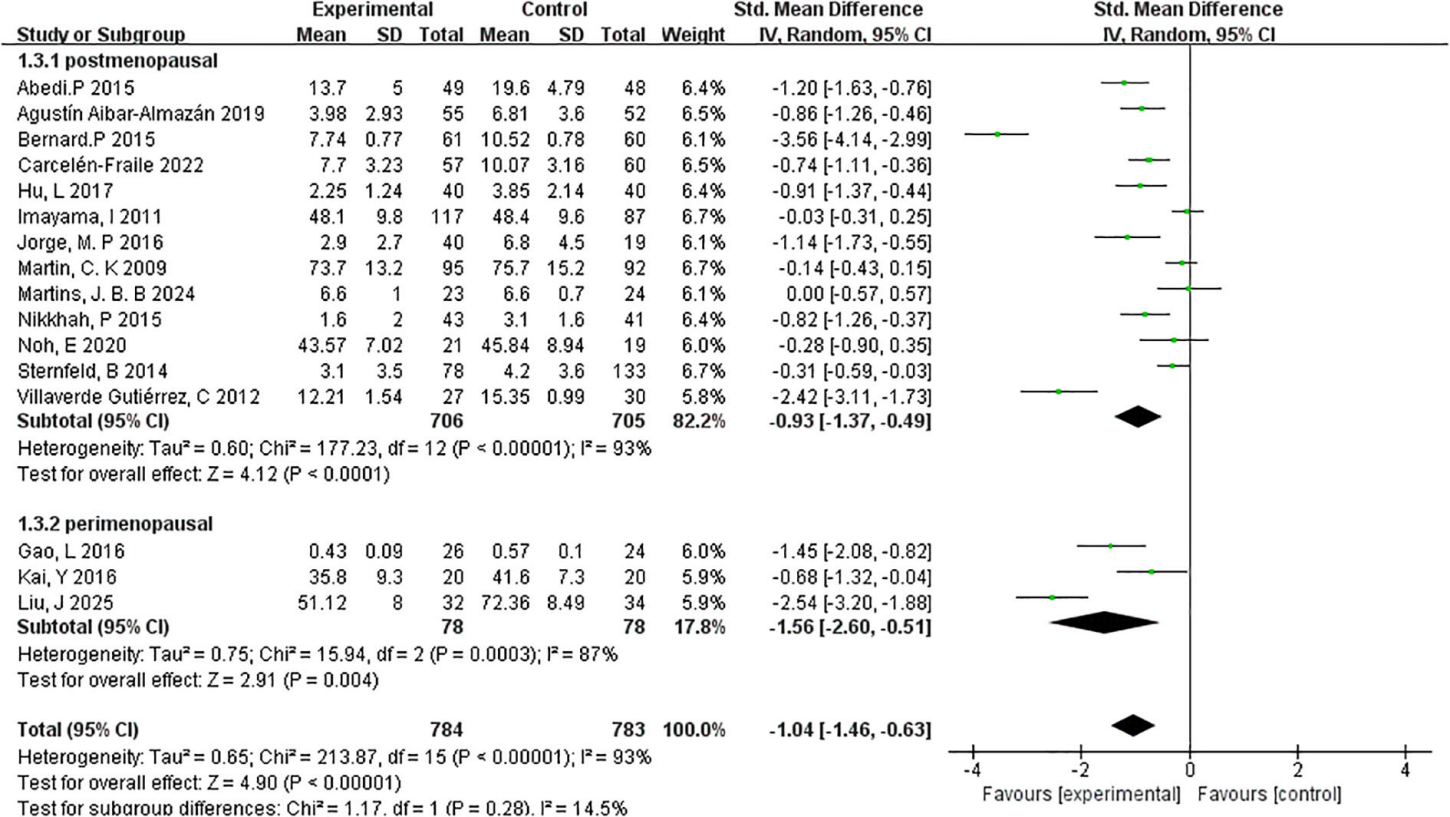
Forest plot of subgroup analyses by menopausal stage.

To explore whether menopausal stage influences the effectiveness of exercise interventions on depressive symptoms, subgroup analyses were conducted for postmenopausal and perimenopausal women (**Figure 7**).

The postmenopausal group included 13 studies with a total of 706 participants. The meta-analysis revealed a statistically significant reduction in depressive symptoms following exercise interventions, with a pooled effect size of SMD=–0.93 (95% CI: –1.37 to –0.49; p < 0.0001). However, heterogeneity within this group was substantial (I²=93%), indicating variability in study populations, intervention types, or assessment tools across the included trials. In contrast, the perimenopausal subgroup comprised 3 studies and 78 participants. This subgroup showed a larger pooled effect size of SMD=–1.56 (95% CI: –2.60 to –0.51; p=0.004), suggesting a more pronounced response to exercise interventions during the perimenopausal stage. Heterogeneity within this subgroup was also high (I²=62%), though notably lower than in the postmenopausal group.

These findings suggest that while exercise interventions are effective across menopausal stages, the impact may be more substantial during the perimenopausal phase, potentially due to heightened symptom variability or hormonal fluctuations. Nonetheless, the small number of studies in the perimenopausal subgroup warrants cautious interpretation. Overall, the subgroup analysis by menopausal stage reinforces the broader conclusion that exercise serves as an effective, non-pharmacological strategy for alleviating depressive symptoms among menopausal women.

##### 3.4.4.3 Type of exercise

**Figure 8:**
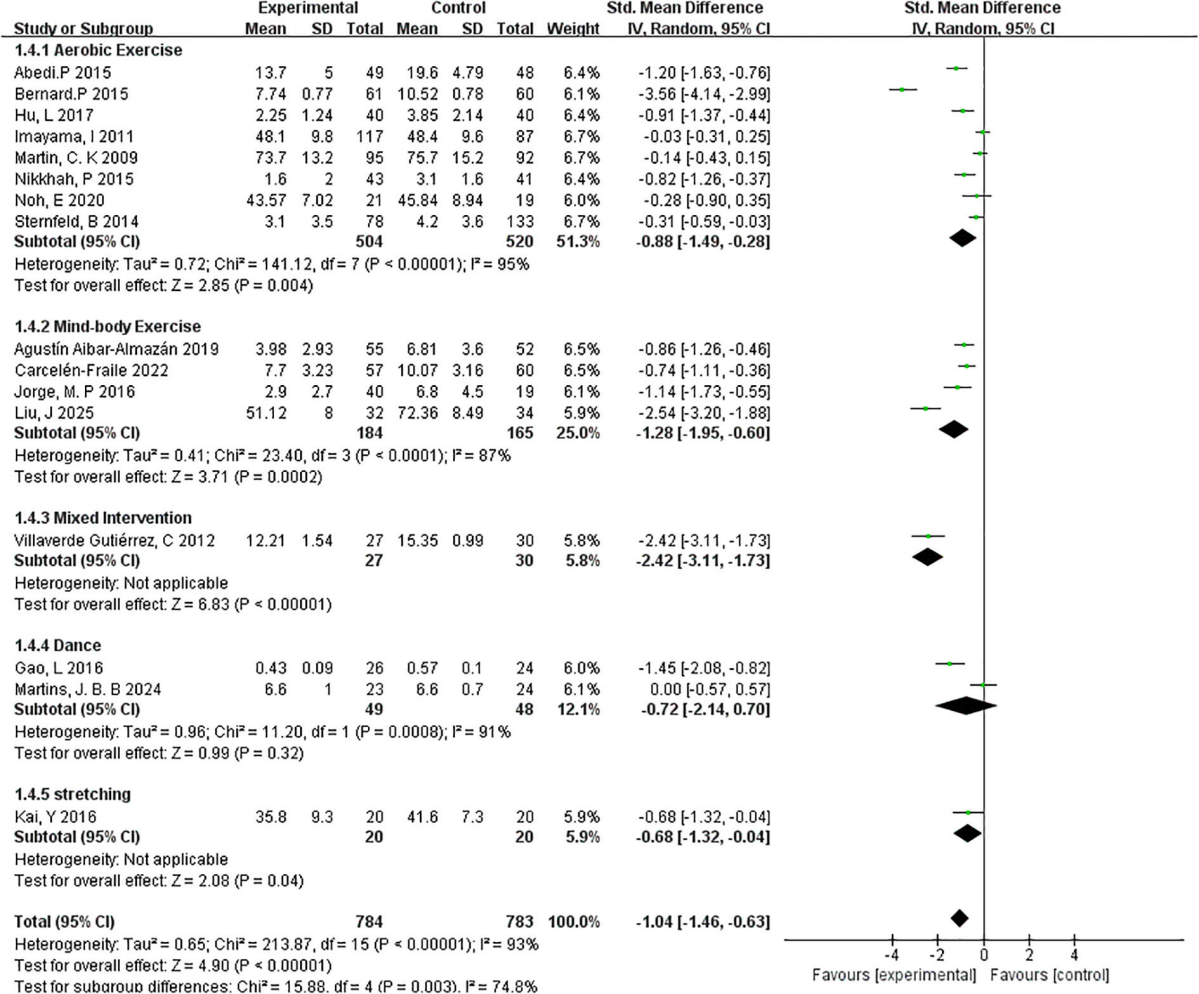
Forest plot of subgroup analyses by type of exercise.

To examine whether the type of exercise modulates the effectiveness of interventions on depressive symptoms, a subgroup analysis was performed across five distinct exercise modalities: aerobic exercise, mind-body exercise, mixed intervention, dance, and stretching (**Figure 8**).

The aerobic exercise subgroup, consisting of 8 studies and 520 participants, showed a statistically significant effect in reducing depressive symptoms (SMD=–0.88, 95% CI: –1.49 to –0.28; p=0.004). However, the heterogeneity was high (I²=95%), indicating considerable variation in intervention protocols, populations, or outcome measurements.

The mind-body exercise subgroup, which included yoga and tai chi (4 studies, 184 participants), demonstrated a more pronounced effect (SMD=–1.28, 95% CI: –1.95 to –0.60; p=0.0002) with slightly lower heterogeneity (I²=87%). These findings suggest that integrating physical movement with mental focus may offer enhanced benefits for mood regulation in menopausal women.

Only one study was classified under mixed interventions, combining elements from multiple exercise modalities. This intervention yielded the largest observed effect size (SMD=–2.42, 95% CI: –3.11 to –1.73; p < 0.00001), but results should be interpreted cautiously due to the single-study basis and lack of heterogeneity estimation. For dance-based interventions (2 studies, 48 participants), the overall effect was not statistically significant (SMD=–0.72, 95% CI: –2.14 to 0.70; p=0.32; I²=91%), likely due to high heterogeneity and limited sample size. Similarly, stretching interventions, examined in a single study (n=20), showed a modest but statistically significant reduction in depressive symptoms (SMD=–0.68, 95% CI: –1.32 to –0.04; p=0.04).

Overall, the results indicate that aerobic and mind-body exercises are the most consistently effective modalities, with mind-body interventions showing particularly strong outcomes. These findings provide preliminary evidence to support the development of targeted exercise programs based on intervention type, although further studies are needed to confirm the efficacy of less commonly used forms such as dance or stretching.

##### 3.4.4.4 Exercise session lengths

**Figure 9:**
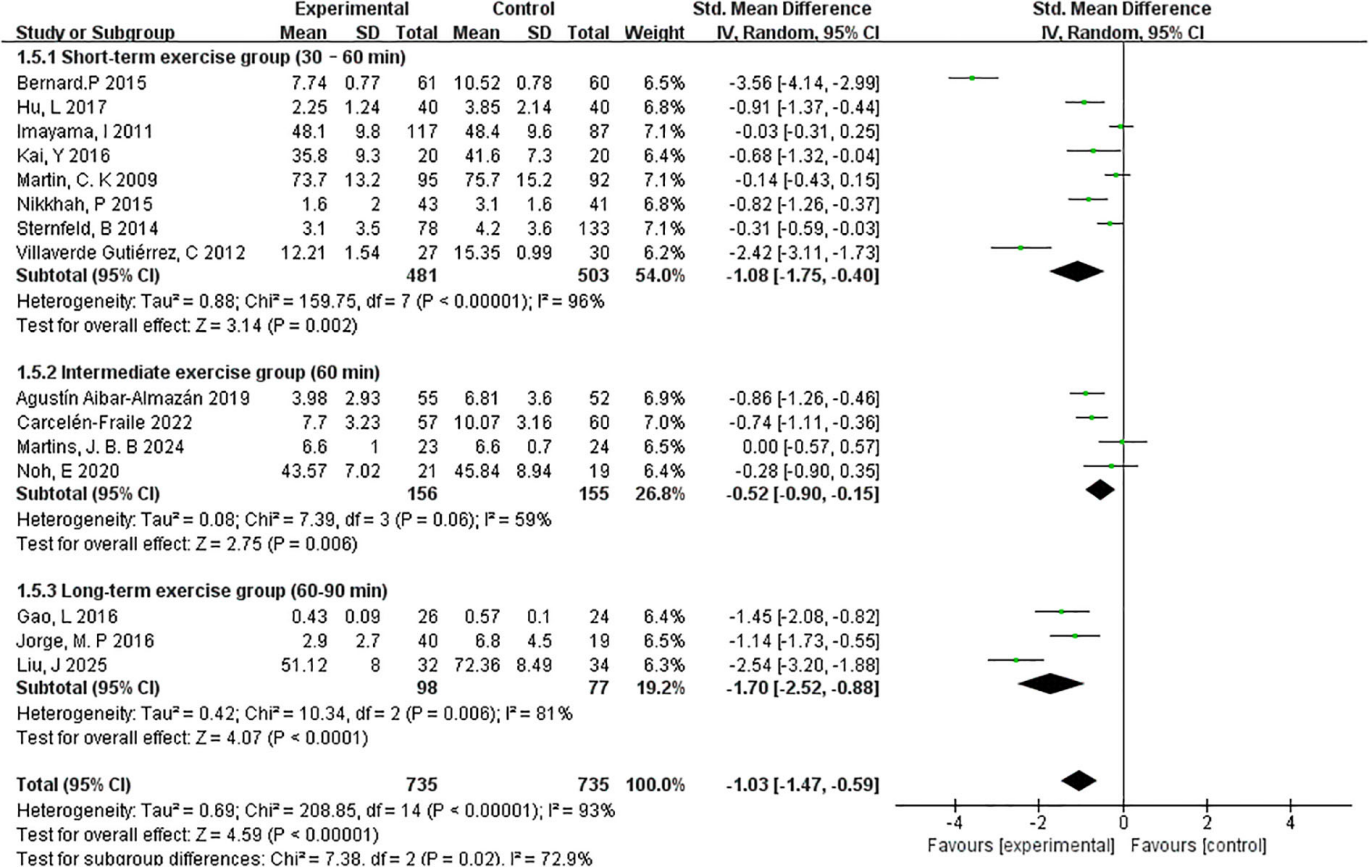
Forest plot of subgroup analyses by exercise session lengths.

To investigate the impact of session duration on the effectiveness of exercise interventions, studies were categorised into three subgroups based on the average length of each exercise session: short-term (30–60 minutes), intermediate (60 minutes), and long-term (60–90 minutes) (**Figure 9**).

The short-term group, which included 8 studies and 481 participants, demonstrated a significant reduction in depressive symptoms with a pooled effect size of SMD=–1.08 (95% CI: –1.75 to –0.40; p=0.002). However, heterogeneity was high (I²=96%), suggesting variability in interventions or participant characteristics across studies.

In the intermediate-duration group (4 studies, 156 participants), the overall effect size was smaller yet still significant (SMD=–0.52, 95% CI: –0.90 to –0.15; p=0.006). This subgroup also exhibited moderate heterogeneity (I²=59%), indicating a more consistent response across studies compared to the short-term group.

The long-term session group (3 studies, 98 participants) yielded the largest pooled effect size among the three subgroups, with SMD=–1.70 (95% CI: –2.52 to –0.88; p < 0.0001). Heterogeneity was also relatively moderate (I²=81%), suggesting that extended session durations may lead to more substantial psychological benefits in postmenopausal women.

Collectively, these findings indicate a potential dose–response relationship between session length and intervention effectiveness, with longer-duration exercise sessions demonstrating the most pronounced effects. However, given the variation in study design and sample sizes, further high-quality trials are warranted to confirm these trends.

##### 3.4.4.5 Duration of the exercise programmes

**Figure 10:**
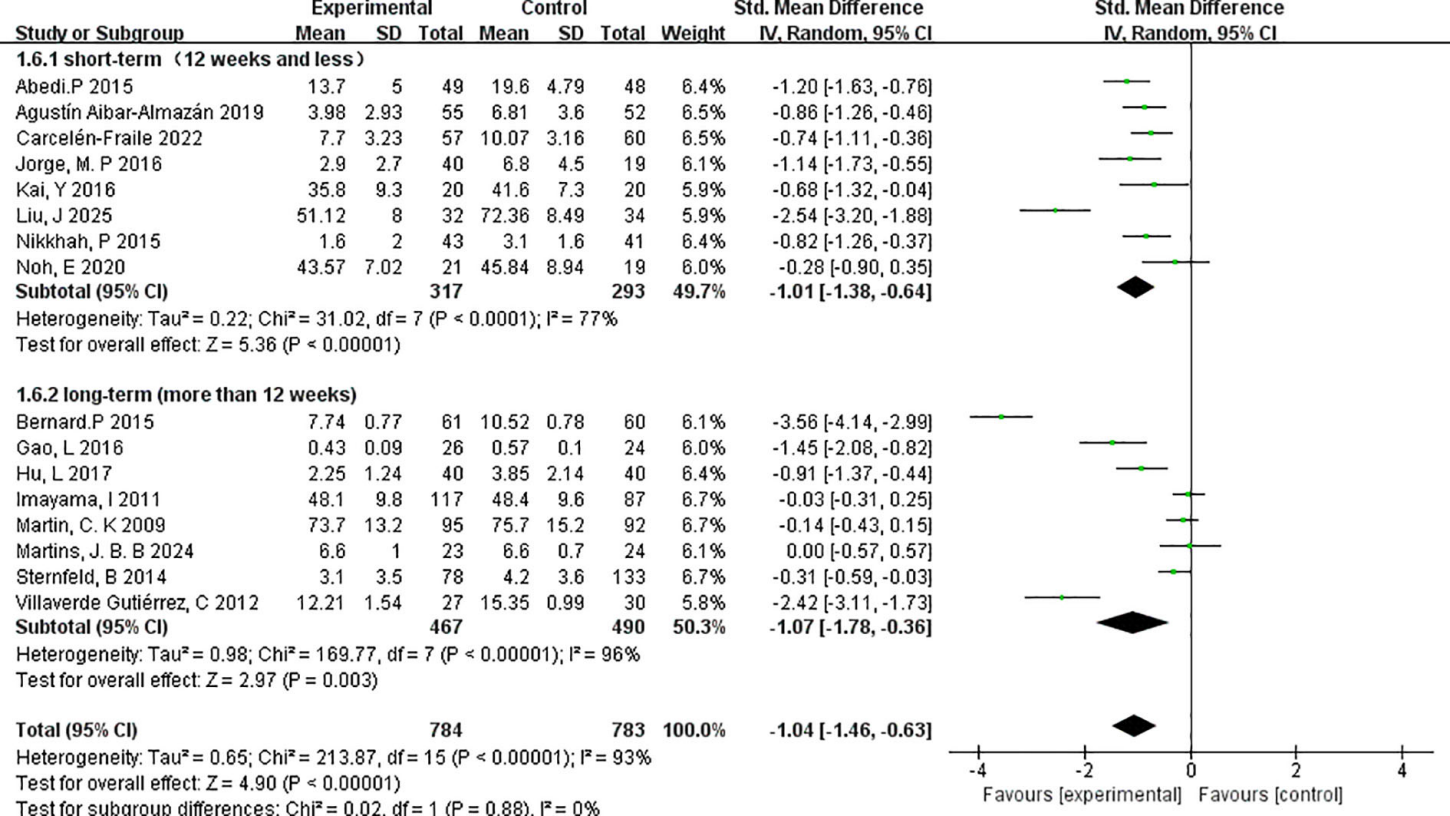
Forest plot of subgroup analyses by duration of the exercise programmes.

To evaluate the influence of intervention length on depressive symptom outcomes, studies were stratified based on the total duration of the exercise programme: short-term (≤12 weeks) and long-term (>12 weeks) (**Figure 10**).

The short-term subgroup, which included 8 studies with a combined total of 317 participants, showed a significant reduction in depressive symptoms following intervention, with a pooled effect size of SMD=–1.01 (95% CI: –1.38 to –0.64; p < 0.00001). Heterogeneity was moderate (I²=77%), indicating some variability across the included studies, possibly due to differences in participant characteristics or intervention types.

The long-term subgroup consisted of 8 studies with 467 participants and yielded a comparable pooled effect size of SMD=–1.07 (95% CI: –1.78 to –0.36; p=0.003). However, this subgroup exhibited higher heterogeneity (I²=96%), suggesting greater inconsistency in the observed effects, potentially due to extended intervention protocols or broader variation in adherence and outcome measurement across longer timelines.

Both short- and long-term interventions demonstrated statistically significant and clinically meaningful effects on depressive symptoms in menopausal women. The similar magnitude of effect across durations suggests that even relatively brief exercise programmes may confer substantial mental health benefits, while longer interventions may yield sustained outcomes. Nevertheless, the elevated heterogeneity in the long-term group highlights the need for more consistent methodological designs in future studies.

## 4 Discussion

This study synthesised evidence from 16 randomised controlled trials to systematically evaluate the impact of exercise interventions on depressive symptoms in postmenopausal women. Meta-analytic results demonstrated a significant reduction in depression levels following exercise (SMD=–1.04, 95% CI: –1.46 to –0.63; p < 0.00001), supporting exercise as an effective non-pharmacological approach for improving mental health in this population. Although high heterogeneity was observed, sensitivity analyses confirmed the robustness and consistency of the overall findings.

A comprehensive subgroup analysis examined five key dimensions: intervention format, exercise type, session duration, total intervention period, and menopausal stage. The analysis revealed that the effectiveness of exercise interventions varies meaningfully across these characteristics. Notably, individually tailored interventions produced greater effects (SMD=–1.43) than group-based formats (SMD=–0.91), suggesting that personalisation and privacy may enhance therapeutic outcomes.

Mind-body exercises, such as tai chi and yoga, yielded stronger benefits than purely physical modalities, potentially due to their emphasis on self-regulation and emotional control. Interventions of longer duration—both in terms of total programme length and single-session length—were associated with more pronounced improvements in depressive symptoms, lending support to a potential dose–response relationship. Furthermore, interventions delivered during the perimenopausal stage produced significantly larger effects than those in the postmenopausal stage, indicating heightened sensitivity to exercise during periods of increased emotional and hormonal fluctuation.

From a physiological perspective, several mechanisms may underlie the antidepressant effects of exercise in menopausal women (35). Exercise has been shown to increase the release of brain-derived neurotrophic factor (BDNF), thereby enhancing neural plasticity and supporting synaptic remodelling in brain regions such as the hippocampus and prefrontal cortex (36, 37). It also contributes to the regulation of the hypothalamic–pituitary–adrenal (HPA) axis, suppression of systemic inflammation, and restoration of neurotransmitter balance, including serotonin (5-HT), dopamine (DA), and norepinephrine (NE) (38). In addition to these neurochemical effects, neuroimaging studies have demonstrated that regular exercise is associated with increased hippocampal volume, improved connectivity within the prefrontal-limbic circuitry, and enhanced cerebral blood flow, all of which are implicated in emotional regulation and cognitive resilience. Furthermore, exercise may stimulate adult hippocampal neurogenesis and modulate the endogenous opioid system, contributing to mood stabilization and stress reduction. These neurobiological and neuroanatomical effects may collectively contribute to improved emotional regulation (39). Moreover, regular exercise can enhance sleep quality, foster greater self-efficacy, and promote a sense of social connectedness—all of which are critical factors in the pathogenesis and mitigation of menopausal depression (40–42).

Compared to earlier studies, which have largely focused on the general efficacy of exercise for menopausal depression, this study addresses a critical gap by systematically examining intervention-specific variables through subgroup analyses (43–45). By categorising studies based on exercise type, frequency, session length, and participant characteristics, this meta-analysis offers novel insights into how intervention structure modulates treatment outcomes. In particular, the analysis of differences between perimenopausal and postmenopausal women, as well as between group-based and individually tailored interventions, provides practical guidance for optimising exercise-based mental health strategies. These findings underscore both the theoretical contribution and clinical relevance of the current study.

Exercise may also interact with pharmacological treatments, potentially enhancing the efficacy of antidepressant medications by synergistically modulating neurotransmitter systems and promoting neurogenesis. However, the benefits of exercise appear to diminish once the intervention is discontinued, suggesting that sustained participation is necessary to maintain its therapeutic effects. Future studies should examine the integration of exercise with medication and strategies to support long-term adherence.

This work is not without limitations. Despite an extensive literature search, the possibility of missing unpublished or grey literature remains, potentially introducing publication bias. Several included trials featured small sample sizes, and variability in depression assessment tools could influence effect estimates despite the use of standardised mean differences for synthesis. In addition, the I² values across analyses were consistently high (>90%), indicating substantial heterogeneity among the included studies. Although a random-effects model and sensitivity analyses were employed, this high heterogeneity inevitably reduces the generalizability of the findings. Moreover, certain subgroup analyses (e.g., perimenopausal women, dance interventions, stretching interventions) included only a limited number of studies and participants, which weakens the reliability of these specific conclusions. Furthermore, confounding factors—such as intervention fidelity, adherence rates, and the concurrent use of psychological therapies—were often inadequately reported or controlled. Another important limitation is that participants’ dietary habits were not considered. Since diet can be influenced by the type of exercise or physical activity practiced, unmeasured dietary differences may have introduced residual confounding across studies. Finally, although multiple subgroup analyses were performed, the limited number of studies within certain subgroups underscores the need for further high-quality, large-scale randomized controlled trials to validate these findings.

Our findings highlight the therapeutic potential of structured exercise for depression in menopausal women. Acknowledging the distinction between exercise and physical activity, future research should explore the dose-response relationship of different exercise modalities and investigate the potential benefits of unstructured physical activity. This would provide a more comprehensive understanding of how varying levels of activity impact mental health.

## 5 Conclusion

This study presents a systematic review and meta-analysis evaluating the efficacy of exercise interventions in alleviating depressive symptoms among postmenopausal women. The findings demonstrate a significant and clinically meaningful effect of exercise, supporting its role as a viable non-pharmacological strategy in managing depression in this population. Subgroup analyses identified several moderating factors influencing intervention outcomes, including exercise format (individual *vs*. group), type (mind-body *vs*. aerobic), session length, total programme duration, and menopausal stage. Notably, interventions that were individualised, incorporated mind–body elements, extended over longer periods, and were delivered during the perimenopausal phase exhibited the strongest effects. These results provide a robust empirical and theoretical foundation for designing personalised, phase-specific, and multi-dimensional exercise programmes for menopausal mental health care.

Despite limitations such as substantial heterogeneity and small sample sizes in certain subgroups, the overall findings were consistent and statistically robust. To refine intervention strategies and elucidate underlying mechanisms, future research should prioritise large-scale, high-quality randomised controlled trials that systematically explore the adaptability and effectiveness of specific exercise characteristics.

In summary, exercise is an effective and promising non-pharmacological strategy for reducing depressive symptoms in postmenopausal women. While further high-quality trials are needed to address heterogeneity and subgroup limitations, the present evidence supports the integration of tailored exercise programmes into menopausal mental health care.

## Data Availability

The original contributions presented in the study are included in the article/**Supplementary Material**. Further inquiries can be directed to the corresponding author.
