# Supplementary material for: Exercise as a therapeutic strategy for depression in menopausal women: a metaanalysis of randomized trials
Source: Front Psychiatry. 2025 Sep 19;16:1641082. doi: 10.3389/fpsyt.2025.1641082 (PMC12492449; doi:10.3389/fpsyt.2025.1641082)
Supplement: Supplementary file 2 [file Table2.pdf]

	Random sequence generation (selection bias)	Allocation concealment (selection bias)	Blinding of participants and personnel (performance bias)	Blinding of outcome assessment (detection bias)	Incomplete outcome data (attrition bias)	Selective reporting (reporting bias)	Other bias
Agustín Aibar-Almazán 2019	+	+	−	−	+	+	?
Carcelén-Fraile 2022	+	+	−	+	+	+	?
Gao, L 2016	+	?	−	?	+	?	?
Hu, L 2017	+	?	−	−	+	?	?
Imayama, I 2011	+	+	−	−	?	?	?
Jorge, M. P 2016	+	+	−	?	+	+	?
Kai, Y 2016	+	+	−	−	+	?	?
Liu, J 2025	+	+	+	+	+	?	+
Martin, C. K 2009	+	?	−	+	+	+	?
Martins, J. B. B 2024	+	+	+	−	+	?	+
Nikkhah, P 2015	+	?	?	−	+	?	?
Noh, E 2020	+	?	−	−	?	?	?
P. Abedi 2015	+	?	+	+	+	+	?
P. Bernard 2015	+	+	+	+	?	?	?
Sternfeld, B 2014	+	+	−	+	+	?	?
Villaverde Gutiérrez, C 2012	+	?	−	?	+	?	?
